# Establishment of an *In Vitro* Assay for Assessing the Effects of Drugs on the Liver Stages of *Plasmodium vivax* Malaria

**DOI:** 10.1371/journal.pone.0014275

**Published:** 2010-12-09

**Authors:** Rana Chattopadhyay, Soundarapandian Velmurugan, Chinnamma Chakiath, Lucy Andrews Donkor, Wilbur Milhous, John W. Barnwell, William E. Collins, Stephen L. Hoffman

**Affiliations:** 1 Sanaria Inc., Rockville, Maryland, United States of America; 2 Walter Reed Army Institute of Research, Silver Spring, Maryland, United States of America; 3 CDC/NCID/Malaria Branch, National Center for Infectious Disease, Centers for Disease Control and Prevention, Chamblee, Georgia, United States of America; The George Washington University Medical Center, United States of America

## Abstract

*Plasmodium vivax* (Pv) is the second most important human malaria parasite. Recent data indicate that the impact of Pv malaria on the health and economies of the developing world has been dramatically underestimated. Pv has a unique feature in its life cycle. Uninucleate sporozoites (spz), after invasion of human hepatocytes, either proceed to develop into tens of thousands of merozoites within the infected hepatocytes or remain as dormant forms called hypnozoites, which cause relapses of malaria months to several years after the primary infection. Elimination of malaria caused by Pv will be facilitated by developing a safe, highly effective drug that eliminates Pv liver stages, including hypnozoites. Identification and development of such a drug would be facilitated by the development of a medium to high throughput assay for screening drugs against Pv liver stages. We undertook the present pilot study to (1) assess the feasibility of producing large quantities of purified, vialed, cryopreserved Pv sporozoites and (2) establish a system for culturing the liver stages of Pv in order to assess the effects of drugs on the liver stages of Pv. We used primaquine (PQ) to establish this assay model, because PQ is the only licensed drug known to clear all Pv hepatocyte stages, including hypnozoites, and the effect of PQ on Pv hepatocyte stage development *in vitro* has not previously been reported. We report that we have established the capacity to reproducibly infect hepatoma cells with purified, cyropreserved Pv spz from the same lot, quantitate the primary outcome variable of infected hepatoma cells and demonstrate the inhibitory activity of primaquine on the infected hepatoma cells. We have also identified small parasite forms that may be hypnozoites. These data provide the foundation for finalizing a medium throughput, high content assay to identify new drugs for the elimination of all Pv liver stages.

## Introduction

*Plasmodium vivax* (Pv) is the second most important malaria parasite, which infects humans. Recent data indicate that the impact of Pv malaria on the health and economies of the developing world has been dramatically underestimated [1], [2]. The number of clinical cases globally due to Pv infection has been estimated to be between 80–300 million per year [3] out of 250–500 million total estimated clinical malaria cases per year caused by all human malaria parasites [4]. Although Pv infection is believed to cause benign disease in comparison to infection caused by *P. falciparum*, some recent studies reported severe symptoms and signs, including death, due to Pv infection [5], [6]. It is also reported that severe symptoms in Pv infection may be due to the infection with multidrug resistant parasites [7], [8].

Pv has a unique feature in its life cycle. Uninucleate sporozoites (spz), after invasion of human hepatocytes, either proceed to develop into tens of thousands of merozoites within the infected hepatocytes or remain for a long time as dormant hypnozoites [9], [10]. The factors, which lead invaded spz in the hepatocytes to take the hypnozoite route, are not known. The primary attack of Pv malaria generally occurs within 10–20 days of exposure to spz-infected mosquitoes. However, activation of hypnozoites after the primary attack causes additional blood stage infections called relapses. Some studies have suggested that the parasites causing relapses are clonally identical to the parasites causing the first primary attack [11], [12]. However, more recently, studies using microsatellite markers identified from the Pv genome sequence showed that relapse infections often result from activation of heterologous hypnozoites [13], [14]. Regardless, for successful control of Pv, complete clearance of hypnozoites is required. At present due to non-existence of any precise biomarker, distinction between hypnozoites and mere slow growing liver stage parasites is not defined at all. Primaquine is the only drug currently available that is known to be effective in killing hypnozoites [15], although nothing is known about its mechanism of action. Primaquine can cause severe hemolytic toxicity in glucose 6-phosphate dehydrogenase (G6PD) deficient subjects and thus screening for G6PD deficiency must be done before administration. Also, as the G6PD status of the unborn fetus cannot be determined easily, primaquine is not prescribed to pregnant women infected with Pv. Tafenoquine, which is an 8 aminoquinoline with a much longer half-life than primaquine is under development [16], but has the same toxicity in G6PD-deficient individuals. Furthermore, unless primaquine is taken with food, it causes significant gastrointestinal disturbance. Therefore, there is an urgent need to develop more drugs, which could effectively control infection by Pv. One of the obstacles to develop effective drugs against Pv pre-erythrocyte stages is the lack of standardized, medium to high throughput hepatocyte based assay platforms or any small animal model of Pv spz infection. Pv hepatocyte stages can be produced *in vitro* by infecting monolayers of primary human hepatocytes [17], HepG2-A16 human hepatoma cells [18], and HC-04 human hepatocytes [19]. It has been quite difficult to reproducibly use these cell culture based platforms for screening new drugs against liver stages, because it has been difficult to obtain adequate quantities of Pv spz, and each time Pv spz are obtained they are from a different strain of Pv or a different infection of non-human primates with the same strain, in other words, a different lot of Pv spz.

At Sanaria Inc., we are developing an attenuated *P. falciparum* whole spz vaccine, the PfSPZ Vaccine. In order to manufacture the PfSPZ Vaccine we have developed methods for aseptic production of *P. falciparum* gametocytes and *Anopheles sp.* mosquitoes, aseptic feeding and maintenance of the infected mosquitoes, and efficient mass extraction of spz from mosquitoes. Most relevant for our work on Pv spz, we have developed methods for purification, formulation, vialing, and cryopreservation of spz [20].

We undertook the present pilot study to, (1) assess the feasibility of producing large quantities of purified, vialed, cryopreserved Pv spz using the technology platform developed at Sanaria Inc. and (2) establish a system for culturing the liver stages of Pv in order to assess the effects of drugs on the liver stages of Pv. We used primaquine (PQ) to establish this assay model, because PQ is the only licensed drug known to have an effect on hepatocyte stage development including clearance of hypnozoites and secondly, unlike with *P. berghei* [21], *P. cynomolgi* and *P. knowlesi* [22], the effect of PQ has not been evaluated on Pv hepatocyte stage development *in vitro*.

## Materials and Methods

### Pv spz

A chimpanzee at the Yerkes Primate Center, Emory University, Atlanta, GA was infected with Pv (India VII strain) in order to have a source of Pv gametocytes. This strain of Pv was earlier adapted to infect and grow in various new world monkeys and chimpanzees [23]. Multiple batches of *Anopheles dirus* mosquitoes were fed on blood from the infected chimpanzee, and it was documented at Center for Disease Control & Prevention (CDC), Atlanta, Georgia that the mosquitoes were infected. The protocol entitled “Induction of *Plasmodium vivax*, *P. malariae* and *P. ovale* infections in chimpanzees to obtain large volumes of parasites for malaria vaccine studies” was approved by the IACUCs at both CDC and Emory University. All *P. vivax* infected chimpanzees were under the supervision of an attending accredited veterinarian, followed twice or thrice weekly by blood smear and treated with anti-pyretic and anti-malarial medications as determined by the attending veterinarian to be appropriate to ameliorate fever, symptoms and to cure the infections. Pv spz-infected mosquitoes were transported to Sanaria Inc. in Rockville, Maryland under controlled and secured conditions. At Sanaria, spz were extracted from infected mosquitoes by dissection of their salivary glands and passing the glands back and forth through a 26½ G needle fitted to a 1 mL syringe. Following extraction, Pv spz were purified from mosquito salivary gland material contamination and either used for infecting HepG2-A16 cells *in vitro* or vialed and cryopreserved in liquid nitrogen vapor phase (LNVP).

### Immunofluorescence based identity test for Pv spz

Purified Pv spz were suspended in PBS containing 2% bovine serum albumin (BSA). 10 µL of this suspension, containing 2000 Pv spz was spotted on each of the 12 wells of immunofluorescence slide (Erie Scientific). Slides were air-dried at room temperature and stored at −80°C in aluminium foil wraps. A monoclonal antibody (mAb), NVS3 [24], against the Pv circumsporozoite protein (PvCSP), was serially two-fold diluted starting at 1∶500 (2 µg/mL) using PBS containing 2% BSA. 20 µL of each of the mAb dilution was added to air-dried spots containing Pv spz and incubated at 37°C in a humid chamber for 1 hour. Slides were washed three times, five minutes each, in a glass trough filled with PBS on a laboratory rocker platform. 20 µL Aleax Fluor 488 Goat anti-mouse IgG was added to each well of the slides and incubated for 1 hour at 37°C in a humid chamber. After washing three times, five minutes each, in a glass trough filled with PBS on a laboratory rocker platform, slides were mounted with cover slips using Vectashield mounting medium. Slides were examined with a fluorescent microscope using a FITC filter.

### HepG2-A16 cell culture

Human hepatoma cells, HepG2-A16, were grown in chambers of 8-well LabTek tissue culture slides (Nunc) using Minimum Essential Medium (MEM) supplemented with 10% fetal bovine serum (MEM-10) at 37°C and 5% CO_2_. In each well of the LabTek slide 20,000 HepG2-A16 cells were seeded one day before infection with Pv spz.

### Infection of HepG2-A16 cells with Pv spz and establishment of Pv hepatocyte stage culture

Medium was discarded from the wells of LabTek slides in which 20,000 HepG2-A16 cells were seeded. The cell monolayer in each well of the LabTek slides was infected with 25,000 Pv spz in 50 µL MEM-10 medium. Slides were incubated at 37°C and 5% CO_2_ for 3 hours after which Pv spz suspension was aspirated off from each well. After three washes with 300 µL MEM-10 medium in each well each time the slides were incubated with 300 µL MEM-10 medium in each well at 37°C and 5% CO_2_ for 3 days with daily change of medium. Another set of slides with 20,000 HepG2-A16 cells in each well were similarly infected with 50,000 Pv spz and maintained in culture for nine days with daily change of medium. Infection rate is defined by the percentage of spz that developed into liver stage parasites expressing PvCSP.

### Infection of HepG2-A16 cells with cryopreserved Pv spz

Vials of Pv spz stored in LNVP were thawed after one year and were used to infect HepG2-A16 cells as described above. The cultures were maintained for 3 days with daily change of MEM-10 medium.

### Immunofluorescence assay (IFA) to elucidate Pv hepatocyte stage parasites

On Day 3 and Day 9 post infection with Pv spz, HepG2-A16 cells in LabTek slides were washed three times with sterile PBS, fixed with chilled methanol for 10 minutes at room temperature, washed again three times with PBS and stored until use with 300 µL PBS in each well at 4°C. Slides from 3 day and 9 day cultures were incubated at 37°C for 1 hour with 100 µL of the anti-PvCSP mab, NVS3 (mAb NVS3, 1∶50 dilution, 20 µg/mL), in each well. Some of the 9 day cultures were also stained with anti-Pv Merozoite Surface Protein-1 (PvMSP-1) ascites, 3F8.A2 (1∶50 dilution), which recognizes late stage Pv schizonts in IFA (J. Barnwell, unpublished) [25]. Secondary conjugate, Alexa Fluor 488 Goat anti-mouse IgG, at 1∶200 dilution in PBS containing 0.02% Evans' blue was added in a 100 µL volume to each well and further incubated for another hour at 37°C. Wells of the slides were washed three times, five minutes each, with PBS, at room temperature on a rocker platform. Slides were mounted with glass cover slips using Vectashield (Hard set) and stored at 4°C overnight. Slides were observed under UV in a fluorescent microscope using FITC filter and the number of hepatocyte stage parasites present in the entire area of each well was counted. The following formula was used to calculate the rate of infection of Pv spz in HepG2-A16 cells- [Mean of number of hepatocyte stage parasites in well/Number of Pv spz added in the well]×100.

### Primaquine (PQ) treatment of Pv hepatocyte stage parasites cultured in HepG2-A16 cells *in vitro*

Establishment of the cell culture for PQ treatment was identical to that described above for 3 day and 9 day cultures of Pv hepatocyte stages. Pv spz were incubated with HepG2-A16 cells for 3 hours. After 3 hours the wells were washed to remove Pv spz in suspension. At that time PQ was diluted from the stock to five 10-fold dilutions from 10.0 µg/mL to 0.001 µg/mL in MEM-10 medium and 300 µL of each dilution was added to triplicate wells. Everyday fresh drug dilutions were prepared in MEM-10 medium and added to the culture. 3 and 9 days post infection of cells with Pv spz and initiation of PQ treatment fixation of cells in chilled methanol followed by labeling with mAb NVS3 was performed as described above for the IFA. The number of hepatocyte stage parasites in the entire area of each well of the slide was counted under fluorescent microscope and the percentage reduction in parasite load in the hepatocytes after 3 days and 9 days of PQ treatment post infection was calculated as- [(M_Medium Control Wells_ − M_PQ treated Wells_)/M_Medium Control Wells_]×100; M =  Mean number of hepatocyte stage parasites.

## Results

### Pv (India VII strain) spz harvest from infected mosquitoes

627 *A. dirus* mosquitoes were processed to harvest spz from their salivary glands. 23.9×10^6^ purified, Pv spz were isolated. A part of this Pv spz harvest was used for setting up assays with HepG2-A16 hepatocytes and part was vialed and cryopreserved in LNVP.

### Pv spz identity test

**Figure 1:**
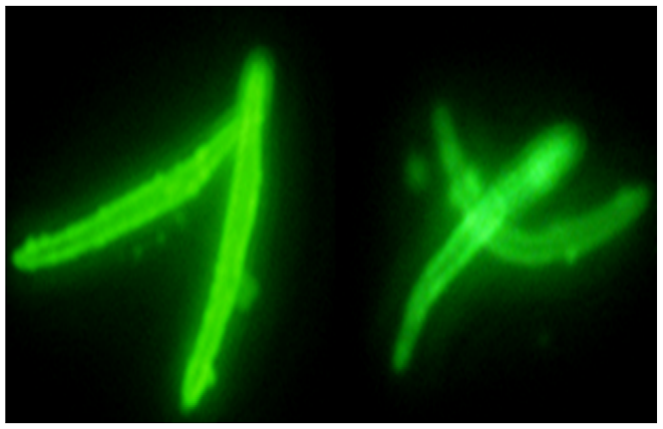
Immunofluorescence Assay. Air dried Pv spz were immunostained with monoclonal antibody against PvCSP (NVS3) at 0.98 ng/mL as described in Materials and Methods. The end point titer is 1∶1,024,000.

An immunofluorescence assay (IFA) using the mAb NVS3 (1 mg/mL) confirmed the identity of the spz as being Pv (Figure 1), because NVS3 mAb reacts exclusively against Pv CSP. Pv spz were found to react with this mAb at a very high dilution, 1∶1,024,000 (0.98 ng/mL).

### Development of hepatocyte stage parasites in Pv spz infected HepG2-A16 cells

**Figure 2:**
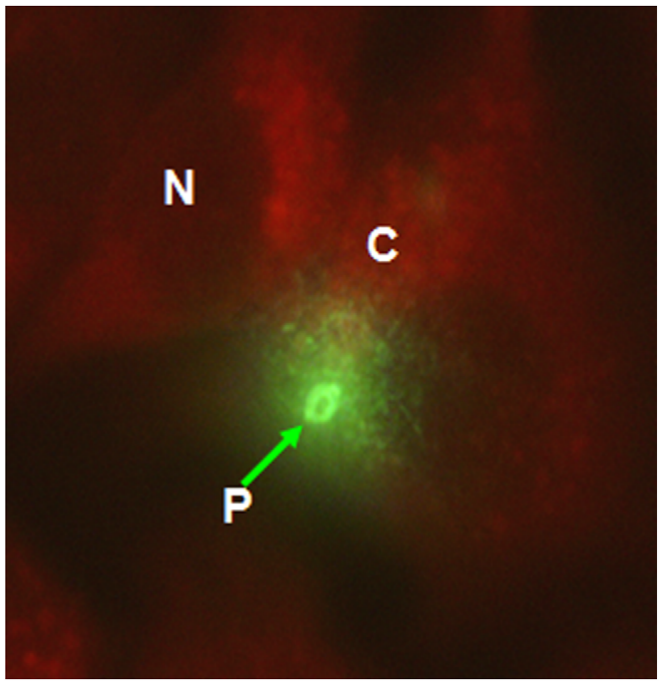
*In-vitro* development of liver stage parasites. 25,000 Pv spz was used to infect 20,000 HepG2-A16 cells. Uninfected spz were washed off after three hrs and cells were maintained for 3 days with daily media change. Cells were fixed and Pv liver stage trophozoites (400 X magnification) were stained with mAb NVS3 against the PvCSP (20 µg/ml). **N**: Nucleus of HepG2-A16 cells, **C**: Cytoplasm of HepG2-A16 cells, **P**: Developing 3 day old Pv hepatocyte stage parasite (trophozoite).

**Figure 3:**
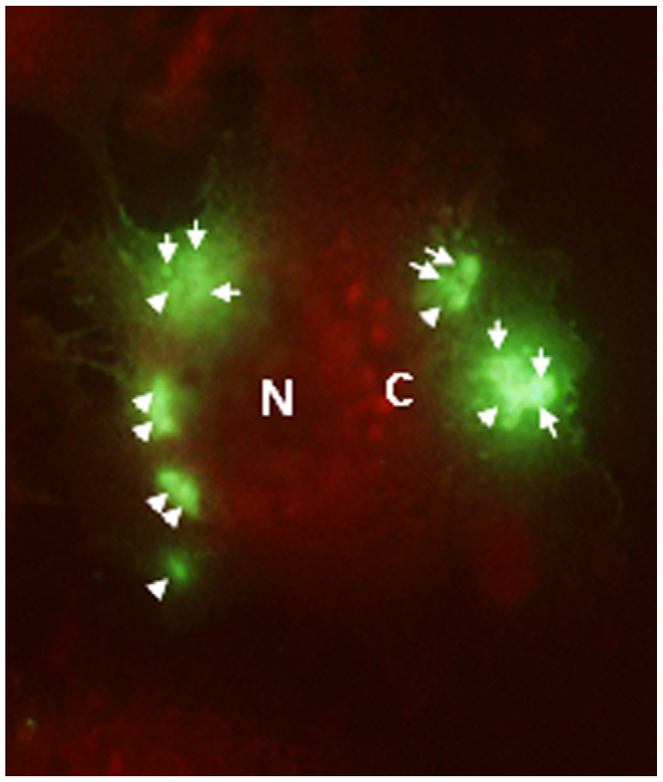
Multiple Pv liver stage parasites were seen in single hepatocyte in a 3 Day culture. HepG2-A16 cells were infected with Pv spz and the liver stage trophozoites were stained with the anti-PvCSP mAb, NVS3. Some HepG2-A16 cells were seen with multiple liver stage parasites (400X magnification). **N**: Nucleus of hepatocyte, C: Cytoplasm of hepatocyte, White Arrows: Individual 3 Day hepatocyte stage Pv.

**Figure 4:**
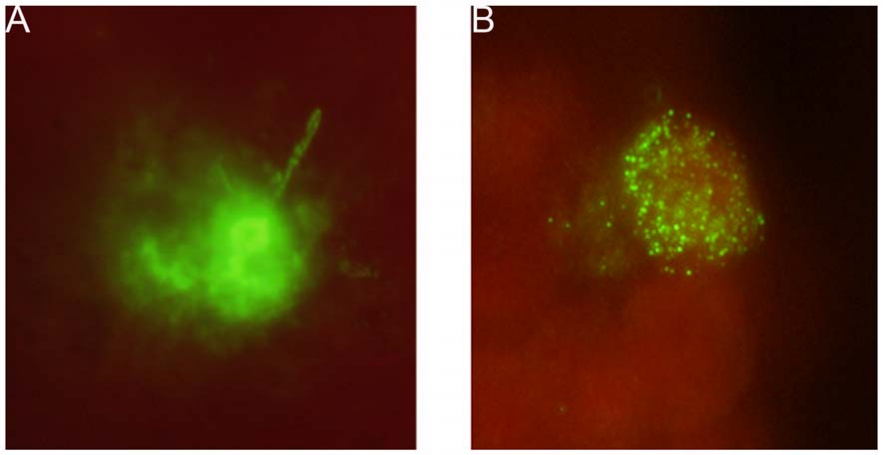
*In-vitro* development of late liver stage schizonts expressing PvMSP1. 20,000 HepG2-A16 cells were infected with 50,000 Pv spz. Three hrs later uninfected Pv spz were washed off and the culture was maintained for 9 days with daily media changes. Mature Pv liver stage schizonts (400 X magnification) in HepG2-A16 cells were stained with (A) the mAb to the PvCSP, NVS3 (20 µg/ml) or (B) with a mAb against Pv merozoite surface protein 1(PvMSP1), 3F8.A2 (1∶50 dilution). As a negative control, uninfected HepG2-A16 cells were incubated with the individual mAbs and labeled secondary antibodies. There was no evidence of staining in these negative control cultures (data not shown).

**Figure 5:**
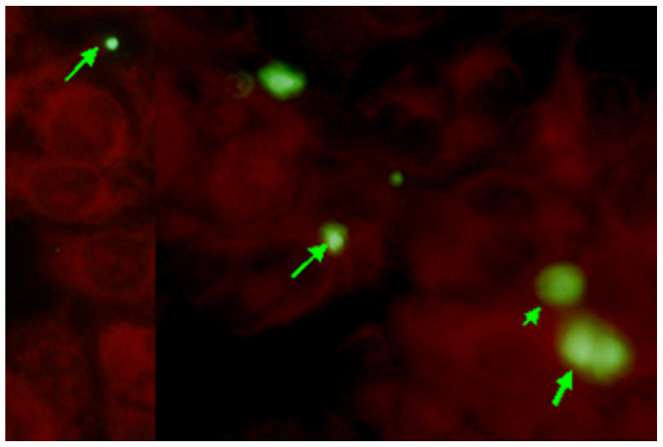
Are these hypnozoites? HepG2-A16 cells were infected, maintained for 9 days as described in Fig 4 and stained with PvCSP mAb. Approximately 10% of the Pv liver stage parasites that expressed PvCSP were similar in size to 3-day trophozoites, and much smaller than the 9-day schizonts. Are these hypnozoites?

25,000 Pv spz were added to 20,000 HepG2-A16 cells in each well, and cultured for 3 days and 50,000 Pv spz were added to 20,000 hepatoma cells in each well, and cultured for 9 days. Figure 2 shows a 3-day-old hepatocyte stage trophozoite of Pv, which reacted strongly with the anti-PvCSP mAb, NVS3, Infection of a single hepatocyte with multiple Pv spz and their subsequent development within the single hepatocyte was also observed (Figure 3). Nine days after infection of HepG2-A16 hepatocytes with Pv spz, large, hepatocyte stage parasites were visible which reacted with mAb NVS3 (figure 4A). These 9 day old parasites also reacted with the anti-PvMSP-1 mAb, 3F8.A2 and developing merozoites in infected hepatocytes were visible (Figure 4B), as has been previously shown with the same mAb [25]. In 9-day cultures, some parasites (generally less than 10%), as small as the parasites seen in the 3 day culture, were also observed to react strongly with the NVS3 mAb (Figure 5). It is possible that these small parasites observed in the 9 day culture were hypnozoites, which did not grow at the same rate as did the parasites that developed to mature late stage hepatocyte stage parasites. The results of a single experiment are shown in Table 1. In the 3-day assay a mean of 458 parasites were identified per well indicating that 1.8% of the 25,000 spz that were added to the well, invaded and developed. In the 9 day assay a mean of 493 parasites (large and small forms) were identified per well indicating that 0.9% of the 50,000 spz that were added to the well, invaded and developed, and stayed identifiable through 9 days of culture.

**Table 1:** *In vitro* development of hepatocyte stage parasites.

Days in Culture	Pv hepatocyte stage Parasites/Well					Infection rate (%)
	Well 1	Well 2	Well 3	Mean	STDEV	
3	461	486	465	457.66	30.14	1.8
9	426	521	492	492.67	28.01	0.9

25,000 Pv spz were added to wells containing 20,000 hepatoma cells and were cultured for 3 days and 50,000 Pv spz were added to wells containing 20,000 hepatoma cells and were cultured for 9 days, and the numbers of early liver stage trophozoites (3 day assay) or late liver stage schizonts (9 day assay) were counted by immunofluorescence microscopy after staining with the anti-PvCSP mAb, NVS3 (20 µg/mL).

### Development of hepatocyte stage parasites in infected HepG2-A16 cells from Pv spz cryopreserved for one year

When Pv spz stored in LNVP for one year were used to infect HepG2-A16 cells, they were found to retain their capacity to invade hepatocytes and transform into hepatocyte stage parasites similar to fresh Pv spz. In a 3-day assay, the percent infection rate with fresh Pv spz was 1.88% and a year later, the percent infection rate with cryopreserved Pv spz from the same lot was 1.95% (Table 2).

**Table 2:** No change in infectivity after cryopreservation of Pv spz.

Date of the Assay	Pv spz Used	Pv Hepatocyte Stage Parasites/Well					Infection Rate (%)
		Well 1	Well 2	Well 3	Mean	STDEV	
Nov 2007	Cryo-preserved	466	510	489	488.3	22.01	1.95
Nov 2006	Fresh	510	439	461	470.0	36.35	1.88

25,000 Pv spz (Fresh or thawed after cryopreserved in LNVP for one year) were added to wells containing 20,000 hepatoma cells and cultured for 3 days. The numbers of early liver stage trophozoites (3 day assay) were counted by immunofluorescence microscopy after staining with the anti-PvCSP mAb, NVS3, as described in Materials and Methods.

### Effect of PQ treatment on hepatocyte stage parasites of Pv *in vitro*

**Figure 6:**
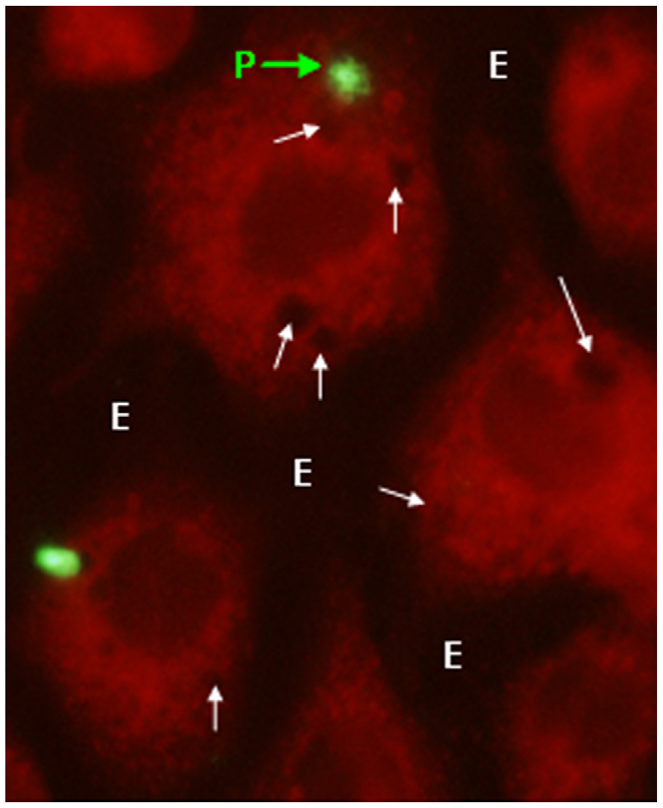
Hepatotoxic changes in HepG2-A16 cells after treatment with high dose primaquine for 9 days. Culturing HepG2-A16 cells with high concentrations of primaquine (10 µg/mL) induced hepatotoxic changes. **P**: Parasite; **White arrows**: Dense bodies in cytoplasm; **E**: Empty spaces due to focal cell loss in the cell culture well.

Pv spz were added to wells containing 20,000 hepatoma cells and cultured for 3 or 9 days. Primaquine (PQ) at 5 different concentrations was added to the wells. The old medium and drug were removed daily and new medium and PQ were added. At the end of the experiment (3 or 9 days) the cells were fixed and the numbers of early liver stage trophozoites (3 day assay) or late liver stage schizonts (9 day assay) were counted by immunofluorescence microscopy after staining with mAb NVS3. Figure 3 (3 day assay) shows a maximum of 40% of reduction in early hepatocyte stage (trophozoites) at 10 ug/mL of PQ whereas Figure 4 (9 day assay) demonstrates a dose response effect of PQ on the development of Pv late hepatocyte stage (schizont) in HepG2-A16 cells. The resulting 50% effective concentration (EC_50_) of PQ after 9 days was between 1 and 0.1 µg/ml. There was no toxicity to the HepG2-A16 cells at any concentration from 0.001 µg/ml to 10 µg/ml in the 3-day assay, and no toxicity to the HepG2-A16 cells at concentrations from 0.001 µg/ml to 1 µg/ml in the 9-day assay. However, in the 9-day assay at the highest dose of PQ (10 µg/mL), significant cytotoxicity of non-infected hepatocytes was observed as marked by focal loss of cells from the culture wells and presence of dense bodies in the cytoplasm of cells (Figure 6). Since the 50% effective concentration (EC_50_) of PQ in this assay was between 0.1 µg/ml and 1 µg/ml, the assay can still be used to demonstrate the effect of PQ on Pv liver stage development. Furthermore, we have subsequently identified a lot of PQ that has the same activity against Pv parasites, but does not have toxicity against the HepG2 cells at 10 µg/mL (Velmurugan S, unpublished).

## Discussion

The goal of malaria eradication [26] cannot be realized without adequate interventions to eliminate infections by all species of malaria parasites that infect humans. Pv causes much less severe disease and death than does *P. falciparum*, but is more widespread globally and infects 80–300 million people every year [3]. Unlike the other major human malaria parasite, *P. falciparum*, there is no *in vitro* cultivation system to produce Pv asexual and sexual erythrocytic stage parasites. Also, like *P. falciparum* there is no small animal model available for Pv. These deficiencies undermine efforts to develop drugs and vaccines against Pv, especially against the pre-erythrocytic stages. Elimination of Pv would be facilitated by development of an easily administered drug that eliminates all liver stage parasites, including hypnozoites, and can be given to the entire population in mass treatment campaigns without concern for life threatening side effects. Identification and development of such a drug would be facilitated by an *in vitro* screening assay.

Herein, we report the first steps toward development of a medium throughput and high content assay to screen for new drugs against Pv liver stages. One of the serious problems with developing such an assay is that it has been extremely difficult to acquire Pv spz for such studies, and that each time a study is done, the study has to be done with spz from a different infection and/or source. This of course limits the capacity to do multiple, reliable comparisons. For the first time we report that cryopreserved Pv spz from the same lot can be used to reproducibly infect hepatoma cells, and that the numbers of 3 day hepatocyte stage parasites are similar in cultures infected with fresh and cryopreserved Pv spz (Table 2). Similar results have now been obtained with cryopreserved Pv spz in the 9 day assay (Velmurugan, S, manuscript in preparation). Being able to produce and vial large single lots of Pv spz that can be used in multiple experiments over time is a first prerequisite to developing a medium to high throughput screening assay.

The next step is to make the assay reproducibly quantitative. The earlier reports on culturing Pv liver stages [17], [18] provided primarily qualitative data that described the ability of primary human hepatocytes or human hepatocyte lines to support the development of hepatocyte stages of Pv and the morphology of these parasites. In the present study, our goal was to measure the rate of infectivity of Pv spz in HepG2-A16 cells and rate of development of invaded Pv spz into hepatocyte stages. We observed that when 25,000 Pv spz were added to 20,000 HepG2-A16 cells and the culture was maintained for 3 days, the overall rate of infectivity was 1.8%, and this was reduced by about 50% to 0.9% when the cultures were maintained for 9 days (Table 1). Our data with Pf spz cultured in HC-04 cells (Chakravarty, S, unpublished), as well as Pv spz cultured in HepG2 cells (Velmurugan, S, unpublished) indicate that about 50% of the spz added to the wells at the outset of the culture are washed out 3 hours later. Thus, of the spz remaining, the overall infectivity is double that which we record for both the 3 and 9 day assays. We think that the difference in numbers of parasites between 3 and 9 day assays is attributable to 1) the fact that some of the 3 day parasites do not fully develop, 2) hepatoma cell death during the period from 3 to 9 days, including cells containing parasites, and 3) replication of the uninfected hepatoma cells in the wells, which makes it more difficult to visualize parasites after 9 days. The infectivity of Pv spz in the HC-04 human hepatocyte line is reported to be 0.041% [19]. HepG2-A16 and HC-04 hepatocyte lines differ biologically, but both of them support development of hepatocyte stages of Pv. The difference in the results from Thailand with HC-04 cells [19] and our results in regard to rate of infectivity in these two lines could be due to the inherent capacity for infection of the two cell lines. However, other factors could have been responsible for the different infectivity rates. We used a single strain (India VII), which has been maintained at CDC since 2001 [23] and fed mosquitoes on infected chimpanzee blood. The Thai group used Pv spz produced from gametocytes infecting people in the field. Another potential factor may be that the purified Pv spz that we use may be associated with higher infection rates, as compared to the unpurified Pv spz used by all other groups. In unpurified Pv spz preparations there may be contaminant molecules of mosquito origin, which compete with Pv spz to bind to the hepatocytes, but purification of Pv spz from these mosquito components might allow Pv spz to attach to and invade hepatocytes more efficiently and at higher rate.

Next we showed, for the first time, that our hepatocyte stage culture system could be used to assess drugs against Pv liver stages *in vitro*. There was a clear dose response when using PQ (Table 3 and 4). Most data suggest that the primary activity *in vivo* when PQ is used to eliminate infected hepatocytes is by a metabolite of primaquine, not by the parent compound. This is probably the reason why such large quantities of primaquine (EC_50_ of 0.1 to 1.0 µg/mL) were required, even for the 9-day assay. Work is in progress in an attempt to isolate the active metabolites of primaquine (Colin Ohrt, personal communication) and we anticipate assessing its activity in the future.

**Table 3:** Dose dependent effect of primaquine on the development of 3 day hepatocyte stage parasites.

PQ Dose (µg/mL)	Pv Hepatocyte Stage Parasites/Well					Reduction in Liver stage Parasites (%)
	Well 1	Well 2	Well 3	Mean	SD	
0.0	461	426	486	457.67	30.14	-
0.001	458	423	485	455.33	31.09	0.51
0.01	431	448	476	451.67	22.72	1.30
0.1	412	443	408	421.00	19.16	8.01
1.0	401	363	349	371.00	26.91	18.93
10.0	291	276	312	293.00	18.08	35.97

25,000 fresh Pv spz were added to wells containing 20,000 hepatoma cells and were cultured for 3 days with medium alone or in the presence of different concentrations of primaquine (PQ). The numbers of early liver stage trophozoites (3 day assay) were counted by immunoflourescence microscopy after staining with the anti-PvCSP mAb (NVS3) as described in Materials and Methods.

**Table 4:** Dose dependent effect of primaquine on the development of 9 day hepatocyte stage parasites.

PQ Dose (µg/mL)	Pv Hepatocyte Stage Parasites/Well					Reduction in Liver Stage Parasites (%)
	Well 1	Well 2	Well 3	Mean	STDEV	
0.0	521	465	492	492.67	28.01	-
0.001	467	483	512	487.33	22.81	1.20
0.01	416	427	478	440.33	33.08	10.75
0.1	301	253	289	281.00	24.98	43.00
1.0	183	142	163	162.67	20.50	66.90
10.0	71	96	43	70.00	26.51	85.80

50,000 fresh Pv spz were added to wells containing 20,000 hepatoma cells and were cultured for 9 days in medium alone or in the presence of different concentrations of primaquine (PQ). The numbers of late liver stage schizonts (9 day assay) were counted by immunoflourescence microscopy after staining with the anti-PvCSP mAb, NVS3 as described in Materials and Methods.

In the 9-day assay 10 µg/mL inhibited parasite development by 86% while in the 3-day assay there was only a 36% reduction at 10 µg/mL. The total cumulative dose of PQ is important for complete elimination of liver stages of Pv *in vivo* [15], [27]. The greater effect of PQ observed in the 9-day assay as compared to the 3-day assay may have been due to the cumulative effect of PQ over 9 days as compared to only 3 days. Future studies will assess the kinetics of activity of PQ and other drugs by looking at the results after 3 to 9 days of treatment. At 10 µg/mL for 9 days, hepatotoxicity was observed, as has been seen in cultures of *P. cynomolgi* and *P. knowlesi* hepatocyte stages in primary rhesus monkey hepatocytes [22]. This would be a limiting variable in these cultures. However, subsequent work with a different lot of primaquine has shown much less cytotoxicity (Velmurugan, S, unpublished).

An ideal drug for elimination of Pv will be effective against hypnozoites. Hypnozoites or dormant hepatocyte stages of Pv were observed *in vivo* and possibly in HepG2-A16 cells after infection with Pv spz [9], [18], [28]. Identifying and characterizing hypnozoites is a major emphasis of our research program. Thus, the small forms expressing PvCSP that we have identified in the 9-day cultures intrigue us. Much work will be needed to determine if these are truly hypnozoites.

We have established the capacity to reproducibly infect hepatoma cells with Pv spz, quantitate the primary outcome variable of infected hepatoma cells and demonstrate the inhibitory activity of primaquine on the infected hepatoma cells. We have also identified small parasite forms that may be hypnozoites. We are now moving to a 96-well format that will use less Pv spz/well and less time to quantitate infected hepatocytes, automated read outs, establishment of the effect of other drugs on the cultures and characterization of the small forms. With these improvements, we anticipate a medium throughput assay can be developed in near future that can be used to screen for new drugs against Pv hepatocyte stages.
